# Frequent FGFR1 hotspot alterations in driver-unknown low-grade glioma and mixed neuronal-glial tumors

**DOI:** 10.1007/s00432-021-03906-x

**Published:** 2022-01-11

**Authors:** Sophie Engelhardt, Felix Behling, Rudi Beschorner, Franziska Eckert, Patricia Kohlhof, Marcos Tatagiba, Ghazaleh Tabatabai, Martin U. Schuhmann, Martin Ebinger, Jens Schittenhelm

**Affiliations:** 1 Department of Neuropathology, Institute of Pathology and Neuropathology, University Hospital of Tuebingen, Eberhard Karls University of Tuebingen, Calwerstr. 3, 72076 Tuebingen, Germany; 2 Department of Neurosurgery, University Hospital of Tuebingen, Eberhard Karls University Tuebingen, 72076 Tuebingen, Germany; 3 Center for Neuro-Oncology, Comprehensive Cancer Center Tuebingen-Stuttgart, University Hospital of Tuebingen, Eberhard Karls University of Tuebingen, Tuebingen, Germany; 4 Department of Radiation Oncology, University Hospital Tuebingen, Hoppe-Seyler-Str. 3, 72076 Tuebingen, Germany; 5 German Consortium for Translational Cancer Research (DKTK), DKFZ Partner Site Tuebingen, Tuebingen, Germany; 6 Institute for Pathology, Katharinenhospital Stuttgart, Stuttgart, Germany; 7 Department of Neurology and Interdisciplinary Neurooncology, University Hospital Tübingen, Hertie-Institute for Clinical Brain Research, Eberhard Karls University Tübingen, 72076 Tuebingen, Germany; 8 Center for Personalized Medicine, Eberhard Karls University of Tuebingen, Tuebingen, Germany; 9 Division of Pediatric Neurosurgery, Department of Neurosurgery, University Hospital of Tuebingen, Eberhard Karls University of Tuebingen, Tuebingen, Germany; 10 Department Pediatric Hematology/Oncology, Children’s University Hospital, Eberhard Karls University of Tuebingen, Tuebingen, Germany

**Keywords:** FGFR1, Pyrosequencing, Low-grade glioma, Mixed neuronal glial tumor, Immunohistochemistry

## Abstract

**Purpose:**

Low-grade gliomas (LGG) and mixed neuronal-glial tumors (MNGT) show frequent MAPK pathway alterations. Oncogenic fibroblast growth factor receptor 1 (FGFR1) tyrosinase kinase domain has been reported in brain tumors of various histologies. We sought to determine the frequency of FGFR1 hotspot mutations N546 and K656 in driver-unknown LGG/MNGT and examined FGFR1 immunohistochemistry as a potential tool to detect those alterations.

**Methods:**

We analyzed 476 LGG/MNGT tumors for KIAA-1549-BRAF fusion, IDH1/2, TERT promotor, NF1, H3F3A and the remaining cases for FGFR1 mutation frequency and correlated FGFR1 immunohistochemistry in 106 cases.

**Results:**

368 of 476 LGG/MNGT tumors contained non-FGFR1 alterations. We identified 9 FGFR1 p.N546K and 4 FGFR1 p.K656E mutations among the 108 remaining driver-unknown samples. Five tumors were classified as dysembryoplastic neuroepithelial tumor (DNT), 4 as pilocytic astrocytoma (PA) and 3 as rosette-forming glioneuronal tumor (RGNT). FGFR1 mutations were associated with oligodendroglia-like cells, but not with age or tumor location. FGFR1 immunohistochemical expression was observed in 92 cases. FGFR1 immunoreactivity score was higher in PA and DNT compared to diffuse astrocytoma, but no correlation between FGFR1 mutation in tumors and FGFR1 expression level was observed.

**Conclusion:**

FGFR1 hotspot mutations are the fifth most prevailing alteration in LGG/MNGT. Performing FGFR1 sequencing analysis in driver-unknown low-grade brain tumors could yield up to 12% FGFR1 N546/K656 mutant cases.

**Supplementary Information:**

The online version contains supplementary material available at 10.1007/s00432-021-03906-x.

## Introduction

Low-grade gliomas (LGG) and the WHO category of mixed neuronal-glial tumors (MNGT) encompass a broad spectrum of mostly pediatric tumors with usually indolent clinical behavior or long-term epilepsy-associated sequelae (Surrey et al. 2019). Excellent outcome can be achieved through gross total resection. However, cases with incompletely resected tumors may experience a chronic, relapsing course (de Blank et al. 2019). Some recurring tumors have poor response rates with chemotherapy and risks of long-term toxicity of irradiation must be carefully weighed in children (Ater et al. 2012). The designation LGG is not consistently used in pediatric and adult neuro-oncology. LGG in ‘adult-type’ diffuse gliomas is used for isocitrate dehydrogenase (IDH) 1 and 2 mutant tumors or IDH-wildtype gliomas exhibiting molecular features of high-grade gliomas and usually progressing to glioblastoma histology (Komori 2021). In contrast, ‘pediatric-type’ LGG include many circumscribed gliomas and tumor progression is rarely reported. Distinct morphologically defined entities of LGG and MNGT include pilocytic astrocytoma (PA), ganglioglioma (GG), dysembryoplastic neuroepithelial tumor (DNT), polymorphous low-grade neuroepithelial tumor of the young (PLNTY), papillary glioneuronal tumor (PGNT) and rosette-forming glioneuronal tumor (RGNT). Recent advances in genomic discoveries allow for a molecular approach for LGG and MNGT stratification and new entities of so called ‘pediatric-type’ diffuse gliomas based on recurrent alterations have been proposed (Ellison et al. 2019). Diffusely growing gliomas in children and adolescents usually show genetic aberrations within the mitogen-activated protein kinase (MAPK) pathway and are characterized by the absence of isocitrate dehydrogenase (IDH) 1 and 2 hotspot and H3 histone, family 3A (H3F3A) mutations (Lazow et al. 2020). The most frequent single driver alterations in these tumors are a v-Raf murine sarcoma viral oncogene homolog B (BRAF) V600E mutation, a fibroblast growth factor receptor (FGFR)1 alteration, or a v-Myb avian myeloblastosis viral oncogene homolog (MYB) or MYBL1 rearrangement which are all associated with a rather favourable outcome (Qaddoumi et al. 2016; Yang et al. 2018). These driving genetic alterations are usually conserved at tumor recurrence (Lazow et al. 2020). Some MAPK alterations overlap with distinct histological subtypes. For example, the BRAF V600E mutation is frequently seen in GG and pleomorphic xanthoastrocytoma (PXA) but is also sometimes reported in PA and diencephalic diffuse glioma (Ho et al. 2015). Similarly, isomorphic diffuse gliomas frequently show MYBL1 (54%) or MYB (23%) rearrangements but these alterations are also seen in diffuse astrocytomas (DA) (Ellison et al. 2019; Zhang et al. 2013). Other alterations are highly diagnostic for a specific tumor entity. The majority of PA exhibit characteristic KIAA1549-BRAF fusions (Collins et al. 2015) and PGNT show a unique methylation profile and highly diagnostic PRKCA fusions (Hou et al. 2019). In contrast, some alterations encompass a broad spectrum of LGG and MNGT tumors and show age-related distribution patterns (Ryall et al. 2020). Especially, FGFR alterations are observed in several tumor entities in up to 7% of neoplasms (Helsten et al. 2016). In brain tumors, FGFR1 alterations are not restricted to tumor grade or a specific age group (Bale 2020).

Four receptors (FGFR1 to FGFR4) and 18 ligands (fibroblast growth factors) have been discovered in humans and have an important role in cell growth, differentiation and neovascularization (Bale 2020; Katoh and Nakagama 2014). Upon ligand binding, FGFR dimerizes and phosphorylates intracellular kinase domains (for example Tyr724 and Tyr760 in FGFR3) thus activating several important druggable pathways including Ras/Raf/MEK and PI3K-Akt (Katoh and Nakagama 2014; Nelson et al. 2018). FGFR1-tyrosinase kinase domain duplications are more prevalent in extracerebellar PA and DNT (Jones et al. 2013; Rivera et al. 2016). In contrast, FGFR1-TACC1 fusion is a distinctive alteration of extraventricular neurocytoma in addition to a small number of other rare FGFR1 and FGFR3 fusions (Sievers et al. 2018). Additional frequent reported alterations are the oncogenic FGFR1 tyrosinase kinase domain hotspot mutations N546 and K656 (Lew et al. 2009). These mutations have been reported in midline and extracerebellar PA (Jones et al. 2013), in posterior fossa PA with widespread oligodendroglial features (Sievers et al. 2020), in DNT (Surrey et al. 2019; Rivera et al. 2016) and in RGNT (Lucas et al. 2020). Furthermore, they are also observed in diffuse midline gliomas in addition to H3 K27M mutations (Schüller et al. 2021). A recent study associated FGFR1 mutations in low-grade glioma with increased risk for intracranial bleeding (Ishi et al. 2020). In experimental murine NF1 models, a co-occurring FGFR1 hotspot mutation confers an additional growth advantage in LGG (Fisher et al. 2021). These hotspot mutations are of great interest, because FGFR alterations can be targeted by inhibitors including the multi tyrosine kinase inhibitors ponatinib, lucitanib and nintedanib (Porta et al. 2017). Some of these FGFR inhibitors are currently under investigation in phase I/II targeted trials for advanced solid tumors (Voss et al. 2019; Touat et al. 2015).

Taken together, the reported FGFR1 N546 and K656 hotspot mutations show a significant overlap in LGG and MNGT entities, are driven by altered MAPK signalling and represent potential diagnostic as well as predictive markers for clinical trial inclusions and eventually clinical management. Open questions addressed in this study include (i) the actual frequency of FGFR1 N546 and K656 hotspot mutations in an unselected collection of low-grade brain tumors independent of their histological designation and (ii) the potential screening role of FGFR1 immunohistochemistry to identify FGFR1 alterations in such tumors.

## Material and methods

### Biological specimen

The formalin-fixated, paraffin-embedded samples were obtained from patients undergoing surgery for brain tumors between 2000 and 2018 at the University Hospital Tübingen. The study was authorized by the respective ethics board (number 708/2017BO2). Histological diagnosis, molecular typing and grading for each tumor sample were performed according to the current WHO classification of CNS tumors (Louis et al. 2016). Tumor location, gender, survival, tumor status (primary/progression), NF1 status and patient age were retrieved from the clinical records.

**Fig. 1:**
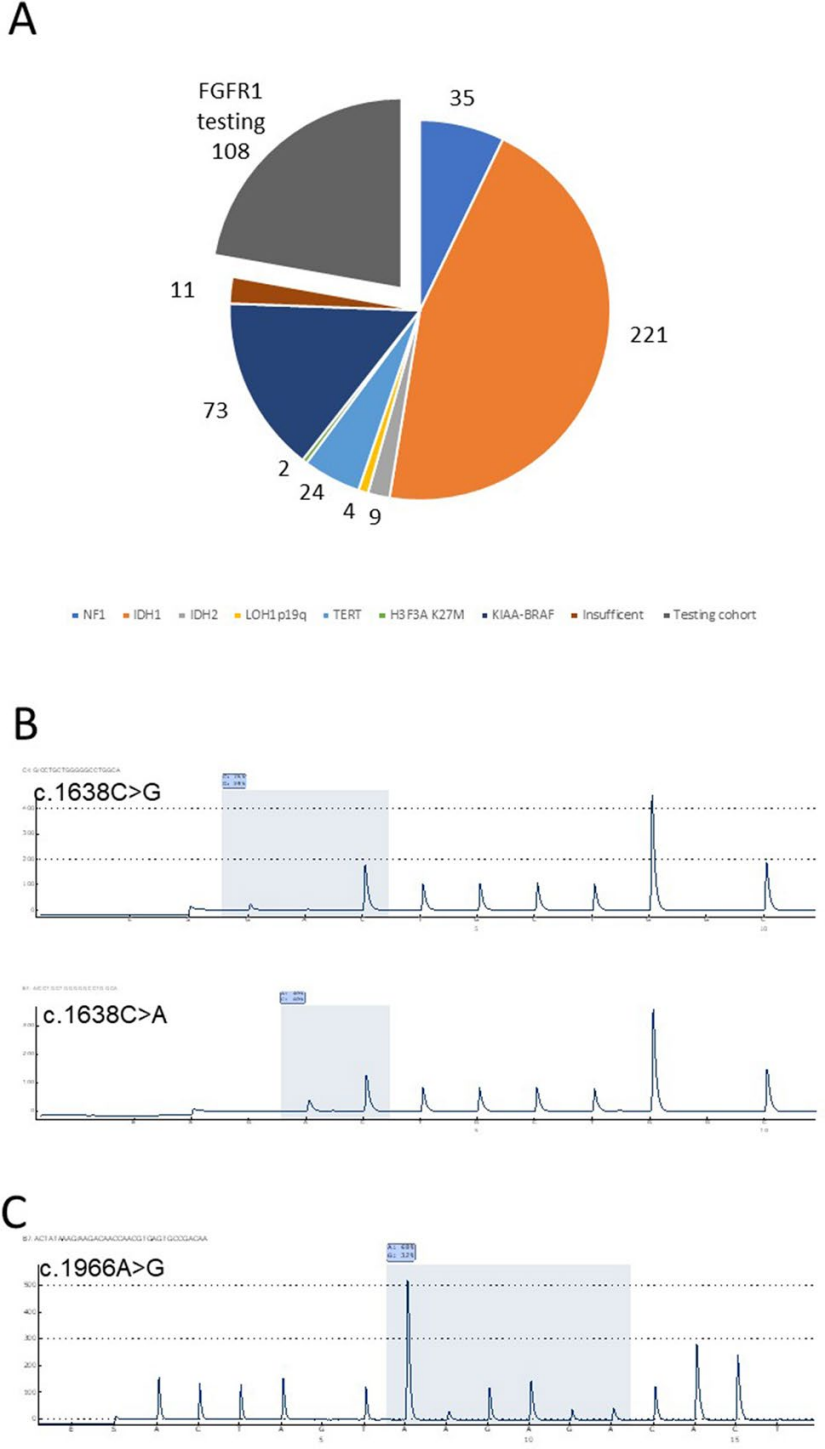
**A** Distribution of Non-FGFR1 mutations across 476 tumors. Sample numbers are provided on top for each slice. 108 samples without known mutation were selected for FGFR1 testing. **B** Representative pyrograms for FGFR1 N546K mutation (five cases with c.1638C > A and four with c.1638C > G). **C** Representative pyrogram for FGFR1 K656E mutation (four cases with c.1966A > G)

681 tumors were listed after initial search from the pathology records for LGG and MNGT diagnosis and cases were reviewed. Eleven samples were excluded, because the remaining archived tumor tissue was too small for immunohistochemistry or molecular analysis. 476 tumors remained after checking for consent of scientific use of samples (Fig. 1A). Based on available data from clinical records or additionally performed molecular analysis as outlined below, the following 368 cases with one or more non-FGFR1 alteration were identified: Confirmed neurofibromatosis type 1 in 35 cases, IDH1 mutation in 221 cases, IDH2 mutation in nine cases, 1p/19q codeletion in four cases without and in 112 cases with IDH1/2 mutation, TERT promotor hotspot mutation in 24 cases without and in 21 cases with IDH1/2 mutation, H3F3A K27M mutation in two cases and KIAA1549-BRAF fusion in 73 cases. Table 1 summarizes the frequency of mentioned alterations for initial tumor diagnosis. Because FGFR1 alterations usually do not occur in the context of these mutations, the subsequent FGFR1 analysis was restricted to the remaining cases without known driver-mutation (Picca et al. 2018). The final FGFR1 sequencing and staining study cohort consisted of 108 samples (45 female and 63 male, mean age 22.8 range 0.6–71 years, Fig. 1A). Epidemiologic details of FGFR1 cohort are shown in Table 2. In 94 cases, the primary tumor and in 20 cases the recurrent tumor was used for analysis because either the primary tumor was not available or not sufficiently DNA could be extracted.

**Table 1:** Molecular results and frequencies of mutations across diagnosis from full cohort

Diagnosis	Samples	IDH1		IDH2		TERT		BRAF-fusion		LOH1p/19q		NF1		K27M		FGFR1	
DA	153	110	71.90%	3	1.96%	23	15.03%	1	0.65%			1	0.65%			1	0.65%
DESCRIPTIVE	10					2	20.00%	1	10.00%	1	10.00%	2	20.00%	2	20.00%		
DIA/DIG	2																
DNT	27							1	3.70%			1	3.70%			4	14.81%
GG	16																
ODG	104	94	90.38%	5	4.81%	17	16.35%			102	98.08%						
MIXED GLIOMA	18	17	94.44%	1	5,56%	3	16.67%			13	72.22%						
PA	138							70	50.72%			31	22.46%			5	3.62%
PXA	4																
RGNT	4															3	75.00%
All samples	476	221	46.43%	9	1.89%	45	9.45%	73	15.34%	116	24.37%	35	7.35%		0.42%	13	2.73%

Details of FGFR1 sequencing subcohort are shown in Table 2

*DA* diffuse astrocytoma, *DIA/DIG* desmoplastic infantile astrocytoma/ganglioglioma, *DNT* dysembryoplastic neuroepithelial tumor, *GG* ganglioglioma, *ODG* oligodendroglioma, *PA* pilocytic astrocytoma, *PXA* pleomorphic xanthoastrocytoma, *RGNT* rosette-forming glioneuronal tumor

**Table 2:** Epidemiological data of samples used for comparative FGFR1 pyrosequencing and immunohistochemistry

Diagnosis	*N* cases	Mean age (yr.)	Min age (yr.)	Max age (yr.)	Gender F/M	Primary/Recurrent
DA	16	35.3	4	71	6/10	15/1
DIA/DIG	2	0.7	0.6	0.75	1/1	2/0
DNT	26	15.2	1	43	13/13	19/7
GG	15	27.9	3	59	5/10	14/1
ODG	2	4.5	4	5	0/2	2/0
PA	37	21.4	1.5	60	14/23	30/7
PXA	4	20.5	6	51	3/1	3/1
RGNT	4	37	11	58	1/3	3/1
DESCRIPTIVE	2	24.5	3	46	2/0	0/2

*DA* diffuse astrocytoma, *DIA/DIG* desmoplastic infantile astrocytoma/ganglioglioma, *DNT* dysembryoplastic neuroepithelial tumor, *GG* ganglioglioma, *ODG* oligodendroglioma, *PA* pilocytic astrocytoma, *PXA* pleomorphic xanthoastrocytoma, *RGNT* rosette-forming glioneuronal tumor. *F* female, *M* male, *yr* year

### Molecular diagnostics

IDH1/2, H3F3A, ATRX, BRAF and LOH1p/19q analysis in the Tübingen cohort was performed as described previously (Ebrahimi et al. 2016). Briefly, the IDH R132H and H3F3A K27M mutational status was first determined by immunohistochemistry. All grade II/III tumors lacking the R132H mutation and grade IV samples aged below 55 years were further examined by direct pyrosequencing of the relevant exons for IDH1 and two hotspot mutations. Cases with ATRX loss and IDH1/2 wildtype status or cases with midline location were sequenced for H3F3A K27 and G34 mutations. Diffuse low-grade gliomas without IDH1/2 mutation were sequenced for BRAF and TERT promotor mutations (Koelsche et al. 2013). Loss of heterozygosity (LOH 1p/19q) was examined in IDH1/2 mutant tumors with ATRX retention using 5 tetranucleotide markers for each chromosomal region. Allele signal intensity of each tumor sample was always compared with the corresponding allele band of the blood control sample of each corresponding patient. BRAF fusion analysis was performed using fusion-transcript-specific PCR after RNA was extracted from FFPE samples using the RNeasy FFPE Kit (Qiagen) (Gierke et al. 2016). Methylation classification analysis on the EPIC platform was retrieved from cases enrolled in the MNP2.0, or PTT trials in five cases (Illumina, Carlsbad, California, USA) and evaluated as previously described (Capper et al. 2018), Tumors were classified with an established brain tumor classifier V11b4 (www.molecularneuropathology.org). Classifier scores with a probability greater 0.9 were taken as indicative for the respective methylation class.

### FGFR1 pyrosequencing

Using a BlackPREP FFPE kit (Analytik Jena, Germany), DNA was extracted from the microdissected tumor tissue according to the manufacturer’s instructions. Tissue was selected from regions on paraffin blocks that presented sufficient (> 50%) tumor content in microscopy. The region around FGFR1 c.546 was amplified with the following primers: FGFR1 c.546 -forward, 5′-CGGACGCAACAGAGAAAGACTT-3′ and FGFR1 c.546 -reverse biotinylated primer, 5′-[BIO]CCCAGATCCCGAGATAACACA-3′. For c.656 we used FGFR1 c.656 -forward, 5′-ACGGGACATTCACCACATC-3′ and FGFR1 c.656 -reverse biotinylated primer, 5’-[BIO]CACCCCACTCCTTGCTTC-3′. In all cases, the estimated sizes of the amplification products detected corresponded to the predicted sizes.

Pyrosequencing was performed on the Pyromark Q24 system according to the manufacturer’s instructions (Qiagen, Hilden, Germany). For pyrosequencing we used starting primer FGFR1 (c.546 5′-AAGCATAAGAATATCATCAA-3′ c.656 5′- CATTCACCACATCGACT-3′) with dispensation orders’GACTGCTGGC’ for c.546 and ‘ACTAGTAAGAGACACT’ for c.656. Pyrograms were analyzed with the PyroMarkQ24 software (Version 2.0.7 Build 3) and a level of 5% for relative light units for variant detection was applied. FGFR1 positive cases were confirmed by repeated pyrosequencing.

### FGFR1 immunohistochemistry

Staining was performed on the Benchmark IHC/ISH (Ventana Medical Systems) after several optimization rounds for tissue pretreatment, antigen demasking and antibody dilution. FGFR1 protein expression was detected by immunohistochemistry using a polyclonal antibody raised against phospho-FGFR1 (Tyr653, Tyr654) of human origin (RRID:AB_1500112, #44-1140G, Thermo-Fisher Waltham, MA, USA). Samples of breast cancer tissue served as positive control. Staining conditions were as follows: FGFR1: OptiView CC1 pretreatment for 32 min, 1:100 dilution, incubated at room temperature for 32 min. All slides were then counterstained with hematoxylin for 2 min. FGFR1 staining intensities were scored as published previously as 0 (no staining), 1 (weak staining), 2 (moderate staining) and 3 (strong staining). For exemplary images see Supplemental Fig. 1. In addition, positive staining in tumors was quantified as follows: 1 (up to 24% tumor cells positive), 2 (25–50% tumor cells positive, 3 (51–75% tumor cells positive) and 4 (more than 75% tumor cells positive). Staining intensities were multiplied with staining quantification into a combined immunoreactivity score (IRS) ranging from 0 to 12.

### TCGA dataset and statistical analysis

Sequencing profiles of patients with LGG of the central nervous system were retrieved from The Cancer Genome Atlas (TCGA) database (https://portal.gdc.cancer.gov/TCGA-LGG). The clinical datasets (mean age 42.9 years, range 14–87 years) consisted of 460 females and 570 males After exclusion of 416 anaplastic tumors, the final set consisted of 128 astrocytomas, 156 oligodendrogliomas and 262 mixed gliomas. Samples were screened for IDH1/2, H3F3A, NF1, BRAF and FGFR1 mutations. Sample-matching TERT promotor mutations and LOH1p/19q status were derived from tabulated data provided by (Ceccarelli et al. 2016). Quantitative and statistical analyses were performed using JMP 14.2.0 (SAS Institute, Cary, NJ, USA). For correlation analyses, we performed unpaired, two-tailed Student’s* t*-test and the Fisher’s exact test to identify possible significant associations or differences between two pairs. Univariate analyses of the different variables were obtained with 95% confidence intervals (CIs). A *p* value < 0.05 was considered as significant.

## Results

### FGFR1 pyrosequencing identifies FGFR1 hotspot mutations in 12% of the LGG/MNGT subcohort of driver unknown cases

108 samples without entity-defining molecular alterations were analyzed for FGFR1 hotspot mutations in pyrosequencing (Fig. 1A) and in 105 cases the signal passed quality checks. We detected 13 (12%) FGFR1 hotspot mutations. A p.N546K mutation was observed in 9/105 tumors (8%, five cases with c.1638C > A and four with c.1638C > G, exemplary pyrograms shown in Fig. 1B). Among the p.N546K mutated cases, three tumors were diagnosed as DNT, four tumors as PA, one tumor as RGNT and the remaining tumor only had a descriptive diagnosis of low-grade neuroepithelial tumor.

A FGFR1 p.K656E mutation was observed in 4/103 tumors (3%, c.1966A > G; Fig. 1C). Two p.K656E mutated tumors were diagnosed as RGNT, one as PA and one as DNT. Taken together, one of the two FGFR1 hotspot mutations was present in 5/37 (13%) PA, in 4/26 (15%) DNT and in 3/4 (75%) RGNT. None of the tumors histologically diagnosed as DA (*n* = 16), GG (*n *= 15), PXA (*n* = 4) or DIA/DIG (*n* = 2) exhibited a FGFR1 p.N546K or p.K656E mutation.

### Clinicopathological overview of FGFR1 mutant cases

**Fig. 2:**
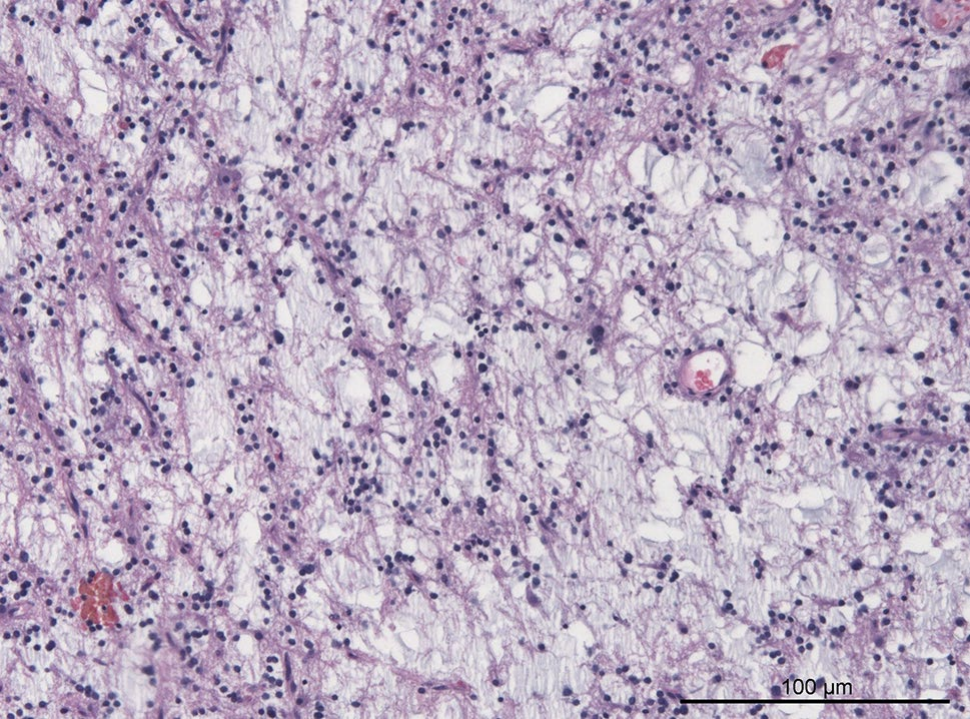
H&E stain of a case with descriptive diagnosis of low-grade neuroepithelial tumor exhibiting a c.1638C > A p.N546K hotspot mutation suggesting DNT as appropriate diagnosis. Reference review pathology also suggested DNT as most likely diagnosis in this case

All FGFR1 mutant cases were reviewed histologically. Common histological features included low cellularity with round nuclei and frequent oligodendroglia-like tumor cells, loosened fibrillary matrix with focal myxoid appearance and low to absent mitotic activity. Vascular proliferations, hemosiderin deposits and calcifications were present in two cases each. Perivascular inflammation was noted in one case. The initial histological diagnosis was confirmed in 11 cases reviewed. One case had a descriptive diagnosis of low-grade neuroepithelial tumor. Detailed histological review of this case showed oligodendroglial-like cells within a loosened myxoid matrix, suggestive of DNT despite absence of floating neurons (Fig. 2). The last case diagnosed initially as DNT showed overlapping features of PA/DNT in review. External reference pathology review (second opinion) in this case also discussed PA/DNT and provided a descriptive diagnosis of low-grade neuroepithelial tumor. Radiological appearance and clinical features also favored DNT in this case and consolidating the initial diagnosis.

Seven of the FGFR1 mutated cases were male, six were female. Six cases were pediatric (3–16 years) and seven cases were adults (23–58 years). FGFR1 mutation was not associated with a distinct tumor location. Seven mutated cases were located supratentorial, six cases were located in the cerebellum. Complete resection could be achieved in seven and subtotal resection in three cases. In the limited follow-up period (2–13 months), three tumors showed progression (one subtotal, one partial resection and one case with biopsy only). In one case, tissue of the recurring tumor was available and the identical FGFR1 hotspot mutation was detected. Presenting symptoms of the tumors included epileptic seizures (*n* = 4), headache (*n* = 5), vertigo (*n* = 2), visual disturbances (*n* = 3) and facial nerve palsy (*n* = 2). There was no significant age difference between the 13 FGFR1 mutant cases combined (mean age 25.8 years) compared to 92 cases without FGFR1 hotspot mutation (mean age 22.6 years, *p* = 0.564). Patients with a FGFR1 p.N546K mutation were slightly but not significantly younger (mean age 19.6 years) and the FGFR1 p.K656E mutant tumors were significantly older at diagnosis (mean age 39.7 years) compared to wildtype cases. In the histological subanalysis, FGFR1 mutated RGNT cases were older (mean age 45.6 years) than the FGFR1 wildtype RGNT case (11yrs), while PA (mean 22.1 vs. 19.6 years) and DNT (mean 14.7 vs. 13.7 years) FGFR1 mutant cases were seen at similar age as FGFR1 wildtype tumors.

### Positive FGFR1 immunohistochemistry does not identify tumors with FGFR1 p.N546 or p.K656 hotspot mutation

Sufficient tissue for FGFR1 staining was available in 106 samples. Expression of FGFR1 was observed in the cytoplasm and membrane of tumor cells and staining intensity was homogenous throughout the tumor tissue. FGFR1 immunohistochemistry staining examples are shown in Supplementary Fig. 1. Absence of staining (score 0) was observed in 16 cases. Weak FGFR1 expression (intensity score 1) was seen in 42 cases (39%), moderate staining intensity (score 2) was present in 30 cases (28%) and strong expression was seen in 18 cases (17%).

Number of positively stained cells varied across divergent tumors. A staining distribution score 1 (less than 25% positive tumor cells) was observed in 3 cases, distribution score 2 in 14 cases, distribution score 3 in 22 cases and distribution score 4 (more than 75% tumor cells positive) in 51 cases.

Combining staining intensity and number of positive cells into an immunoreactive score showed low IR scores (1–4) in 41 cases, intermediate IR scores (5–8) in 36 cases and high IR scores (9–12) in 13 cases.

The FGFR1 IR scores were independent of patient age (*p* = 0.8725) and gender (*p* = 0.554). Primary tumors had nonsignificantly lower FGFR1 IR scores (mean 4.8) compared to tumor recurrences (mean 5.7, *p* = 0.326). FGFR1 IR scores were significantly higher in WHO grade I tumors (mean 5.4, *p* = 0.0053) compared to WHO grade II tumors (mean 2.8). For FGFR1 immunohistochemistry details across histological diagnoses, see Table 3. Interestingly, FGFR1 IR score was significantly higher in PA (mean 6.4, *p* = 0.0002), PXA (mean 7.8, *p* = 0.0101) and DNT (mean 4.8, *p* = 0.0255) compared to DA (mean 2.3).

FGFR1 IR scores in p.N546K or p.K656E mutated tumors were higher (mean 5.8) than in FGFR1 wild-type tumors (mean 4.9. *p* = 0.0370), but results were also not significant in further subgroup analysis after separation for WHO grade or tumor recurrences.

**Table 3:** FGFR1 immunohistochemistry

Diagnosis	*N*	Mean IRS	SD IRS
DA	16	2.3	2.52
DNT	26	4.8	3.70
PXA	3	7.8	0.28
GG	15	4.5	2.89
PA	37	6.4	3.52
RGNT	4	5.6	3.35
Other	7	3.5	4.17

Mean and standard deviation (SD) of immunreactive score (IRS) by histological diagnosis

*DA* diffuse astrocytoma, *DIA/DIG* desmoplastic infantile astrocytoma/ganglioglioma, *DNT* dysembryoplastic neuroepithelial tumor, *GG* ganglioglioma, *ODG* oligodendroglioma, *PA* pilocytic astrocytoma, *PXA* pleomorphic xanthoastrocytoma, *RGNT* rosette-forming glioneuronal tumor

### TCGA dataset analysis

Among 614 sample datasets with LGG histology record, we identified 253 cases with IDH1 and 14 cases with IDH2 mutation. TERT promotor mutations were found in 154, LOH 1p/19q codeletion in 81, NF1 alterations in 10 and a BRAF V600E mutation in two cases. One case each exhibited a KIAA1549-BRAF fusion, a H3F3A K27M mutation and one missense FGFR1 N577K mutation. The FGFR1 mutant case was a 34-year-old male with diagnosis of oligodendroglioma grade II. No follow-up data were available in this case. A cross-check with the COSMIC database (URL: https://cancer.sanger.ac.uk/cosmic/mutation/overview?id=125179833, accessed on 28.10.2021) shows that the FGFR1 N577K mutation has been reported previously in five high-grade gliomas and two RGNT.

## Discussion

A significant subset of low-grade gliomas and mixed neuronal-glial tumors demonstrate ambiguous or overlapping morphological features and diagnostic accuracy of brain tumors can be improved through combining histological and molecular data (Lucas et al. 2020). With the present study we tried to determine the frequency of potentially actionable FGFR1 hotspot mutant tumors in a histological diagnosis-independent subcohort of LGG/MNGT without other known driver mutations. Among 476 tumors with molecular data available, we identified 13 activating FGFR1 p.N546 or p.K656 mutant cases, while 368 tumors exhibited non-FGFR1 alterations. This indicates an overall frequency of 2.7% FGFR1 hotspot mutations in unselected LGG/MNGT cohorts. The most common non-FGFR1 alterations were IDH1/2 mutations (234 cases), KIAA1549-BRAF fusion (73 cases), 24 TERT C228/C250-mutated gliomas and 35 cases with NF1. In our cohort, FGFR1 hotspot mutations are the fifth most occurring genetic alteration in LGG/MNGT tumors. A systematic FGFR1 analysis of these samples after exclusion of these four more frequent mutations would result in 12% FGFR1 N546/K656 mutant cases. The FGFR1 mutations were observed only in tumors diagnosed as pilocytic astrocytoma, dysembryoblastic neuroepithelial tumors and rosette-forming glioneuronal tumor. Therefore, further restricting the FGFR1 sequencing analysis to non-diffusely growing gliomas/MNGT tumors would yield 15% FGFR1 N546/K656 mutant cases after exclusion of cases with BRAF fusion, IDH1/2, NF1 or TERT mutation. We did not find FGFR1 hotspot mutations in our DA or PXA. As seen from the additional TCGA dataset analysis, FGFR1 alterations in DA are rare. In this context, it is noteworthy, that while PA, DNT and RGNT share frequent FGFR1 hotspot alterations they represent epigenetically distinct tumor entities with separate methylation clusters (Capper et al. 2018). In accordance with our data, Qaddoumi et al. reported FGFR1 hotspot mutations only in DNT and diffuse oligodendroglial tumors, while DA instead contained one FGFR1 fusion and two FGFR1 TKD duplications (Qaddoumi et al. 2016). A study analyzing the molecular profile of adult brainstem gliomas reported a surprisingly high FGFR1 mutation frequency in 18% of patients (13/73 cases) (Picca et al. 2018). These mutations were also reported in thalamic and cerebellar tumors in both H3F3A K27M mutated and K27 wild-type tumors. The vast majority of these cases had a high-grade histology, whereas our cohort was intentionally restricted to LGG and MNGT tumors. Both low-grade tumors with H3F3A K27M mutation underwent panel-sequencing and co-occurring FGFR1 mutations could be excluded. Methylation analysis in these cases clearly clustered them together with K27M-mutant pontine gliomas suggesting histological underdiagnosis due to limited sample size. Another integrated molecular analysis of 70 low-grade glioma cases with NF1 tumors revealed 3 FGFR1 hotspot mutation. Such tumors were classified as non-pilocytic astrocytoma based on DNA methylation analysis (Fisher et al. 2021). Except for a single DA case, all NF1 tumors in our initial selection cohort consisted of classical PA, in which no FGFR1 mutations are expected. Three quarters of our RGNT samples were FGFR1 mutated in accordance with a previous publication identifying FGFR1 hotspot mutations in 10/10 RGNTs (Lucas et al. 2020). A previous study reported p.N546K (*n* = 22) or p.K656E (*n* = 8) alterations in all 30 RGNT and additional PIK3CA mutations in 63% and NF1 mutations in 23% of these tumors (Sievers et al. 2019). As expected, the majority (44%) of PA exhibited KIAA1549-BRAF fusions, followed by 12% NF1 mutant and 3% FGFR1 mutant samples. Again this data is in agreement with the suggested frequency of 5% FGFR1 mutations in pilocytic astrocytoma (Collins et al. 2015). While the detection of an FGFR1 alteration in a LGG/MNGT tumor of uncertain subtype may not aid in differential diagnosis between PA, DNT and RGNT, it may help to further narrow the differential diagnosis and exclude certain tumor entities, such as DA and PXA. A study reported on five LGG cases with DNA methylation profiles that did not align with any reference methylation classes but had FGFR1-alterations. In these cases, oligodendroglial-like cells without well-defined patterned nodules, floating neurons, neurocytic rosettes, piloid processes, Rosenthal fibers, or other specific histologic findings, were described (Lucas et al. 2020). Because in two cases a FGFR1 alteration in combination with PIK3CA or PIK3R1 mutation was present, the authors questioned whether such samples may represent a yet-to-be defined tumor class of RGNT outside of the stereotypic location in the fourth ventricle. Furthermore, the FGFR1 hotspots may provide a rationale for targeted FGFR1 treatment (Porta et al. 2017).

As a large center with expertise in pediatric neurooncology the cases referred to us have an inherent selection bias for challenging neurosurgery cases which in turn may impact frequency of FGFR1 alterations. Further limitations of our study include the retrospective nature and archived sample retrieval, differences in the clinical indications for biopsy and restrictions on the amount and type of material available for molecular characterization and restriction of samples to driver unknown LGG/MNGT. Because methylation array tumor clustering is not feasible in such a large cohort, our approach to select cases by histology diagnosis, does not completely exclude cases with potential FGFR1-TACC1 fusions and FGFR1-TKD tandem duplication (Qaddoumi et al. 2016). As this study proposes additional molecular hotspot FGFR1 testing for a subset of driver unknown LGGs, we intentionally restricted our cohort to previously non-informative cases after removing mutually exclusive entity-defining mutations (Picca et al. 2018). However in rare instances, three secondary FGFR1 hotspot mutations have been reported among 364 IDH-mutant gliomas and such cases may have been missed by our methodology (Ahrendsen et al. 2021).

Taken together, our analysis indicates that FGFR1 hotspot mutations are a common event in non-diffusely growing gliomas, especially in PA, DNT and RGNT cohorts, and not associated with a distinct histological pattern further suggesting that MAPK-altered tumors in pediatric and adult samples encompass a broad spectrum of tumors. Performing FGFR1 sequencing analysis routinely in non-diffusely growing driver-unknown low-grade brain tumors could yield up to 15% FGFR1 N546/K656 mutant cases.

## Supplementary Information

Below is the link to the electronic supplementary material.

Supplementary file1 (DOCX 871 KB)
