# Supplementary material for: Frequent FGFR1 hotspot alterations in driver-unknown low-grade glioma and mixed neuronal-glial tumors
Source: J Cancer Res Clin Oncol. 2022 Jan 11;148(4):857–66. doi: 10.1007/s00432-021-03906-x (PMC8930952; doi:10.1007/s00432-021-03906-x)
Supplement: Supplementary file 1 — Supplementary file1 (DOCX 871 KB) [file 432_2021_3906_MOESM1_ESM.docx]

# Supplemental file to “Frequent FGFR1 hotspot alterations in low grade glioma and mixed neuronal-glial tumors.”

Sophie Engelhardt^1^, Felix Behling^2,3^, Rudi Beschorner^1,3^, Franziska Eckert^3,4,5^, Patricia Kohlhof^6^, Marcos Tatagiba^2,3^, Ghazaleh Tabatabai^3,5,7-8^ Martin U. Schuhmann^2,3,9^, Martin Ebinger^10^, Jens Schittenhelm^1,3^

**Correspondence to:**

Prof. Dr. Jens Schittenhelm (ORCID: 0000-0002-9168-6209)

Department of Neuropathology

Institute of Pathology and Neuropathology

University Hospital of Tuebingen,

Calwerstr. 3

D-72076 Tuebingen

Tel.: +49-7071-2982283

Fax: +49-7071-294846

Email: jens.schittenhelm@med.uni-tuebingen.de


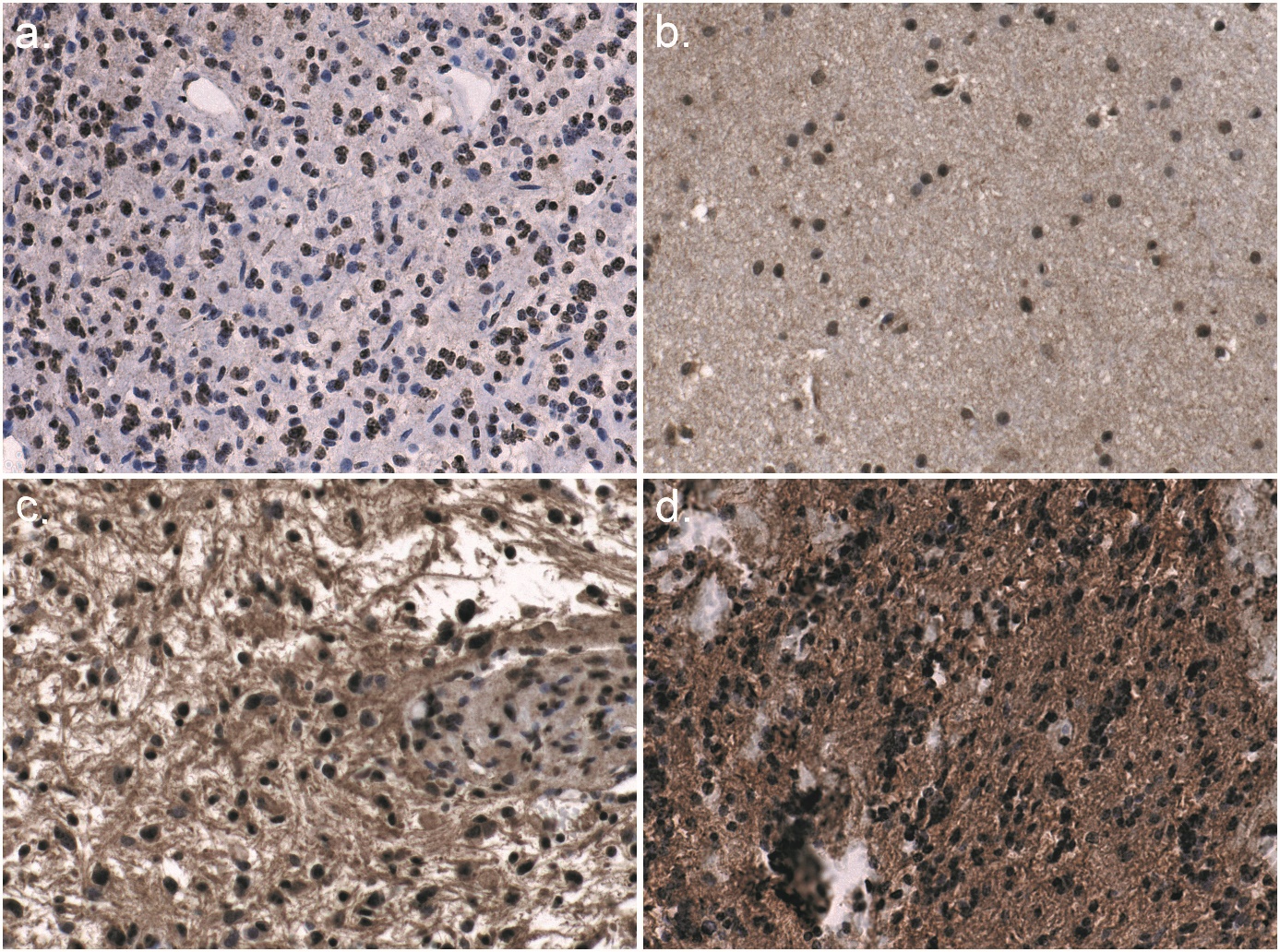


Supplemental Figure: Exemplary FGFR1 staining score: 0: absence of cytoplasmic staining, 1: weak cytoplasmic staining, 2: moderate cytoplasmic staining, 3: strong FGFR1 cytoplasmic staining.
